# A mixture of anatase and rutile TiO_2 _nanoparticles induces histamine secretion in mast cells

**DOI:** 10.1186/1743-8977-9-2

**Published:** 2012-01-19

**Authors:** Eric Y Chen, Maria Garnica, Yung-Chen Wang, Alexander J Mintz, Chi-Shuo Chen, Wei-Chun Chin

**Affiliations:** 1 Bioengineering, University of California at Merced, Merced, CA, USA. 5200 North Lake RD, Merced, CA 95343, USA

**Keywords:** TiO_2 _nanoparticles, mast cell, histamine release, Ca^2+ ^signaling

## Abstract

**Background:**

Histamine released from mast cells, through complex interactions involving the binding of IgE to FcεRI receptors and the subsequent intracellular Ca^2+ ^signaling, can mediate many allergic/inflammatory responses. The possibility of titanium dioxide nanoparticles (TiO_2 _NPs), a nanomaterial pervasively used in nanotechnology and pharmaceutical industries, to directly induce histamine secretion without prior allergen sensitization has remained uncertain.

**Results:**

TiO_2 _NP exposure increased both histamine secretion and cytosolic Ca^2+ ^concentration ([Ca^2+^]_C_) in a dose dependent manner in rat RBL-2H3 mast cells. The increase in intracellular Ca^2+ ^levels resulted primarily from an extracellular Ca^2+ ^influx via membrane L-type Ca^2+ ^channels. Unspecific Ca^2+ ^entry via TiO_2 _NP-instigated membrane disruption was demonstrated with the intracellular leakage of a fluorescent calcein dye. Oxidative stress induced by TiO_2 _NPs also contributed to cytosolic Ca^2+ ^signaling. The PLC-IP_3_-IP_3 _receptor pathways and endoplasmic reticulum (ER) were responsible for the sustained elevation of [Ca^2+^]_C _and histamine secretion.

**Conclusion:**

Our data suggests that systemic circulation of NPs may prompt histamine release at different locales causing abnormal inflammatory diseases. This study provides a novel mechanistic link between environmental TiO_2 _NP exposure and allergen-independent histamine release that can exacerbate manifestations of multiple allergic responses.

## Background

Allergic inflammation is a primary pathological feature of many debilitating diseases [1]. Among the numerous active mediators and cytokines that modulate initiation and progression of allergic inflammation, histamine is distinctly potent [1,2]. Typically, the storage of histamine is restricted to mast cells and circulating basophils [2,3]. The cardinal pathway of histamine release involves the attachment of IgE-bound allergens to high-affinity FcεRI receptors on mast cells and the crosslinking of adjacent IgE molecules by allergens [1,2]. Subsequent receptor clustering leads to a complex cascade of intracellular Ca^2+ ^signaling resulting from increased activity of phospholipase C (PLC), generation of diacylglycerol (DAG) (activating PKC) and inositol 1,4,5-trisphosphate (IP_3_) which mobilizes the ER Ca^2+ ^store and participates in final histamine secretion from mast cells. Activation of histamine receptors (H1, H2, H3 and H4) greatly influences inflammatory responses [2].

Aside from inducing acute allergic inflammatory responses, histamine also mediates chronic phase progression by augmenting the secretion of pro-inflammatory cytokines such as IL-1α, IL-1β, and IL-6 as well as chemokines like RANTES [1,4]. Consequential pathologies are expressed in many systems encompassing ocular, airway, skin and GI tracts [2]. Associated disorders may include asthma, allergic rhinitis, allergic conjunctivitis, atopic dermatitis, urticaria, anaphylaxis and food allergies [1,4,5]. Possible clinical symptoms include itchiness, increased vascular permeability, edema, leukocyte infiltration, bronchoconstriction and mucus hypersecretion [1,4,5]. Therefore, any disturbance to the immunological and/or homeostatic control of histamine release can potentially intensify inflammation leading to health problems.

Recently, numerous epidemiological studies have suggested that pollution associated airborne particulate matter (PM) can aggravate allergic inflammatory responses. Classical examples of allergies indicate that people with asthma and rhinitis are more susceptible to the short term acute effects of particle exposure [6-8]. In line with epidemiological studies, results from animal models also demonstrated that ultrafine particles (one of the major components of PM) can modulate asthmatic responses by exacerbating pulmonary inflammation and airway hyper-responsiveness [9-11]. In an atopic, dermatitis-like, skin lesion mouse model, exposure to titanium dioxide nanoparticles (TiO_2 _NPs) was found to worsen symptoms by elevating proinflammatory molecules in the skin and increasing serum levels of IgE and histamine [12]. At the same time, exposure to environmental tobacco smoke has been found to increase the risks of rhinoconjunctivitis and allergic conjunctivitis [13,14]. Moreover, Kulig et al reported that prenatal and postnatal exposure to environmental tobacco smoke in children (< 3 yrs old) was associated with sensitization to food allergens [15]. Despite NPs' ability to potentiate allergic responses, the role that histamine plays in mediation is not clear. More importantly, whether NPs can directly modulate histamine release from mast cells without allergen sensitization remains elusive.

TiO_2 _NPs have been extensively utilized in the nano-technological and pharmaceutical arenas and are one of the main components in many household commodities and personalized products [16,17]. The enormous annual global production of TiO_2 _broadens the possibilities of occupational and environmental exposures [18]. As a common constituent of PM10, TiO_2 _NPs are widely known for their potential hazardous effects, which manifest biologically via inflammatory responses [19]. TiO_2 _NPs size, surface area and crystalline structure ascribe cellular nanotoxicity [20-23]. In addition to respiratory inhalation, NPs can enter the human body via alternative routes such as: direct penetration through skin, ingestion and injection [24]. Animal studies have shown that intra-tracheally instilled TiO_2 _and other NPs can possibly transmigrate from lung to systemic circulation [25-27]. Upon entering blood circulation, NPs can infiltrate multiple organs, potentially directly stimulating mast cells, a critical effector, exacerbating pathological consequences [24]. As such, TiO_2 _NPs were selected as ideal model particles for our study.

Previously, we have demonstrated that TiO_2 _NPs can trigger a cascade of cytosolic Ca^2+ ^signaling leading to mucin secretion [17]. In the present study, we aim to investigate the impact of NPs on histamine secretion from RBL-2H3 mast cells. We hypothesize that TiO_2 _NPs can directly induce histamine release without prior allergen sensitization via a Ca^2+^-mediated pathway.

## Results

### TiO_2 _NP characterization

**Figure 1:**
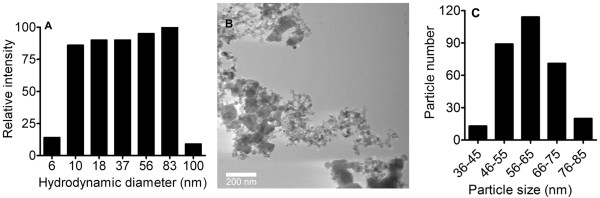
**TiO_2 _NP characterization using DLS and TEM**. (A) DLS assessment of TiO_2 _NP in Hanks' solution revealed a size distribution of ~6 to 100 nm. (B) A representative TEM image of the TiO_2 _NPs with an average diameter of 60 ± 10 nm (n = 300). (C) A bar graph showing the size distribution of TiO_2 _NPs based on TEM images (n = 300).

Dynamic laser scattering (DLS) was used to characterize the TiO_2 _NPs in suspension. The particle size in Hanks' solution had a distribution of ~6 to 100 nm due to minor aggregation while the predominant size was ~83 nm (Figure 1A). Transmission electron microscopy (TEM) provided detailed characterization of TiO_2 _NP size where the mean particle diameter was found to be 60 ± 10 nm (Figures 1B and 1C).

### TiO_2 _NP-Induced [Ca^2+^]_C _increase

**Figure 2:**
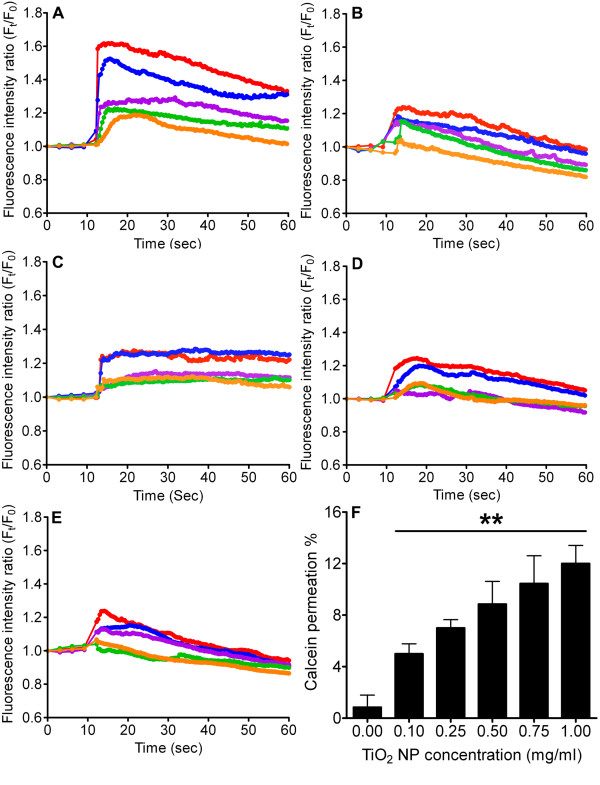
**Measurement of the [Ca^2+^]_C _and calcein leakage after TiO_2 _NP exposure**. (A) RBL-2H3 cells were stimulated with TiO_2 _NPs with concentrations of 0.1 mg/ml (orange), 0.25 mg/ml (green), 0.5 mg/ml (purple), 0.75 mg/ml (blue) and 1 mg/ml (red) in normal Hanks' solution, (B) in Ca^2+^-free Hanks' solution, (C) in the presence of CdCl_2 _(200 μM), (D) nifedipine (10 μM), (E) verapamil (100 μM), and (F) calcein (50 μM) (n ≥ 12, **p < 0.005) (all colors are as depicted in Figure 2A and each line is a representative fluorescence intensity of approximately 200 cells).

We tested whether TiO_2 _NPs could trigger [Ca^2+^]_C _increase by loading RBL-2H3 cells with Rhod-2 AM dye and exposing them to 0.1 mg/ml-1 mg/ml of TiO_2 _NPs. The TiO_2 _concentration range used in our study is consistent with the concentrations found in ambient, nanotechnology industries and existing literatures [17,20,28]. The changes in [Ca^2+^]_C _were measured by monitoring the intracellular fluorescence intensity. Figure 2A shows that 1 mg/ml induced an approximate 160% increase, while lower TiO_2 _NP concentrations (< 0.25 mg/ml) caused a smaller elevation, when compared to untreated cells. The results demonstrated a TiO_2 _NP concentration dependent increase in [Ca^2+^]_C _(Figure 2A).

### Extracellular source of [Ca^2+^]_C _increase

The source of elevated [Ca^2+^]_C _was identified by stimulating RBL-2H3 cells with TiO_2 _NPs in Ca^2+^-free Hanks' buffer with EGTA added to chelate traces of Ca^2+^. Figure 2B demonstrates that TiO_2 _NPs (0.1 mg/ml-1 mg/ml) failed to induce a significant increase in [Ca^2+^]_C_, when compared with the 160% increase observed in normal Hanks' buffer (Figure 2A). Our data suggested that the extracellular Ca^2+ ^pool was the primary source of the observed [Ca^2+^]_C _increase. We then examined if TiO_2 _NPs can induce extracellular Ca^2+ ^influx via membrane channels, in particular L-type Ca^2+ ^channels. Blocking the channels with CdCl_2 _(200 μM) markedly inhibited the rise in [Ca^2+^]_C _by approximately 75% (Figure 2C). Subsequent pre-treatment of cells with nifedipine or verapamil (L-type Ca^2+ ^channel blockers) also hampered the rise in [Ca^2+^]_C _elicited by TiO_2 _NPs (Figure 2D and 2E). Nonetheless, the incomplete blockage of Ca^2+ ^influx with membrane channel blockers suggests a possible Ca^2+ ^leakage through perturbed cell membranes. To confirm that TiO_2 _NPs can instigate membrane disruption, thereby permitting unspecific extracellular Ca^2+ ^entry, a fluorescent calcein dye was used to assess cytosolic leakage. Results showed a dye permeation ratio increase from approximately 5 to 12% with a TiO_2 _NP concentration ranging from 0.1 to 1 mg/ml (Figure 2F).

### Induction of oxidative stress and associated Ca^2+ ^influx

**Figure 3:**
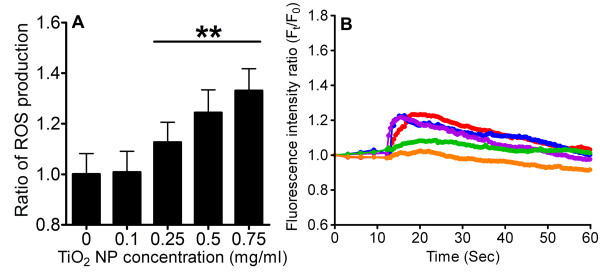
**Measurement of intracellular ROS level and the associated changes in [Ca^2+^]_C_**. (A) RBL-2H3 cells were exposed to TiO_2 _NPs at concentrations 0, 0.1, 0.25, 0.5 and 0.75 mg/ml. The generation of ROS was measured by fluorescence imaging. The level of ROS increased as a function of increasing TiO_2 _NP concentration (n ≥ 50, **p < 0.005). (B) Pretreatment with antioxidant NAC (250 μM) significantly reduced the rise in cytosolic Ca^2+ ^when stimulated with TiO_2 _NPs. Each line is a representative fluorescence intensity of approximately 200 cells and the colors used are consistent with Figure 2A.

Demonstrating possible effects of oxidative stress evoked by TiO_2 _NPs, intracellular ROS formation was investigated. Transient exposure of RBL-2H3 cells to TiO_2 _NPs for 15 min resulted in cytosolic ROS level increase in a dose-dependent manner (Figure 3A). Since oxidative stress can trigger intracellular Ca^2+ ^signaling, cells pretreated with an antioxidant NAC were monitored for changes in [Ca^2+^]_C _upon TiO_2 _stimulation. Our data revealed that NAC notably attenuated the increase in [Ca^2+^]_C _triggered by TiO_2 _NPs (Figure 3B).

### The IP_3_-IP_3 _receptor as a histamine-release pathway

**Figure 4:**
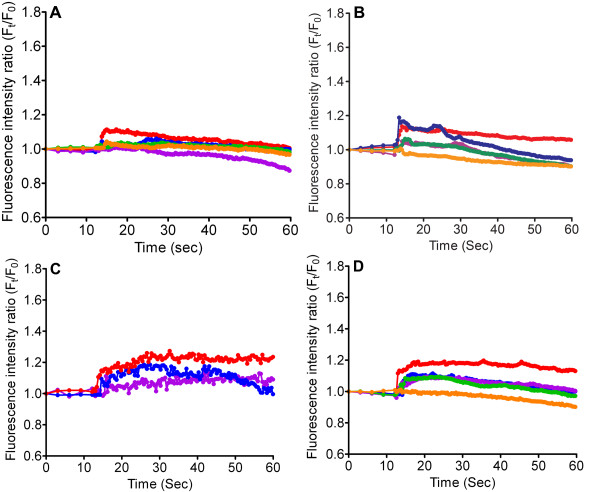
**Measurement of [Ca^2+^]_C _after stimulation by TiO_2 _NPs**. RBL-2H3 cells were stimulated with TiO_2 _NPs with concentrations that vary from 0.1 mg/ml-1 mg/ml, in the presence of (A) U73122 (10 μM), (B) 2-APB (50 μM) and (C) Xestospongin (20 μM) and (D) Thapsigargin (100 nM). The colors used are in accordance with Figure 2A. Each line represents the average fluorescent intensity of more than 200 cells.

To understand the influence of PLC, and the ensuing IP_3_-IP_3 _receptor pathway on cytosolic Ca^2+^, the RBL-2H3 cells were pre-treated with U73122 (PLC inhibitor), 2-APB or Xestospongin (IP_3 _receptor blockers), before being exposed to TiO_2 _NPs. The data revealed a > 75% drop in the [Ca^2+^]_C _after TiO_2 _NP stimulation (Figure 4A-C). Since ER is one of the main intracellular Ca^2+ ^stores, it is highly responsible for the amplification of intracellular Ca^2+ ^signals. Thapsigargin was used to deplete intracellular ER Ca^2+ ^pool and caused a decrease in the [Ca^2+^]_C _after TiO_2 _NP exposure (Figure 4D).

### Ca^2+ ^dependency of TiO_2 _NP-induced histamine secretion

**Figure 5:**
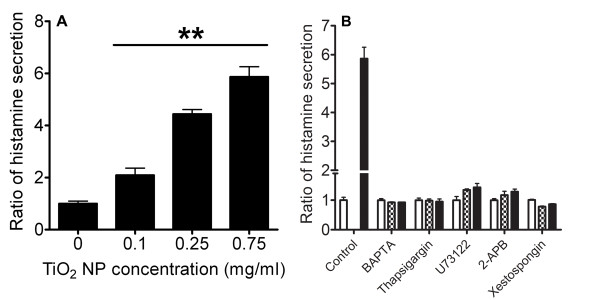
**Measurement of histamine secretion evoked by TiO_2 _NPs**. (A) ELISA quantification of histamine release from RBL-2H3 cells after TiO_2 _NP (0.1 mg/ml-0.75 mg/ml stimulation) in normal Hanks' solution (n ≥ 3, **p < 0.005). (B) Subsequent assessment of histamine secretion from RBL-2H3 cells when pre-treated with an intracellular Ca^2+ ^chelator (BAPTA) and various blockers prior to TiO_2 _NP exposure (n ≥ 3; white, checkboard, and black bars represent 0, 0.5 and 0.75 mg/ml, respectively).

ELISA was used to assess the amount of histamine secreted from RBL-2H3 cells when stimulated with TiO_2 _NPs. Comparing with the control, TiO_2 _NPs increased histamine secretion in a dose-dependent fashion (Figure 5A). Incubation with BAPTA-AM (intracellular calcium chelator), Thapsigargin, U73122, 2-APB or Xestospongin significantly attenuated histamine secretion (Figure 5B). Our data indicated that histamine release is attributed to a high [Ca^2+^]_C _sustained by an external Ca^2+ ^influx and a secondary Ca^2+ ^release from intracellular Ca^2+ ^stores (organelles).

## Discussion

Recently, a growing epidemic of exacerbated allergic inflammatory diseases have been closely linked with the exposure to airborne PM pollution [5,7,8]. Many prior investigations have focused on the hazardous effects of NPs. These reports have primarily been either epidemiological studies that draw correlations between clinical manifestations and exposure to environmental pollutants or immunological studies that examine the release of inflammatory cytokines and subsequent recruitment of immune cells using allergen-sensitized mice with PM or NP insults [7,9,10]. Few studies have systematically investigated the role of histamine secretion from mast cells during NP challenge and its role in mediating allergic inflammation. By using allergen-sensitized animal models, these studies overlooked the NP's potential to directly stimulate histamine release from mast cells without prior allergen sensitization or IgE production. In addition, the associated underlying cellular mechanisms leading to the final degranulation are not clear. TiO_2 _NPs have previously been shown to trigger mucin secretion, pulmonary inflammatory responses and emphysema-like pathology [17,18,29,30]. In this study, we demonstrate that TiO_2 _NPs can directly stimulate histamine release from RBL-2H3 mast cells via a Ca^2+^- dependent pathway.

Allergic inflammatory diseases encompass a multifactorial interplay between many elements/systems; our study focuses on how TiO_2 _NPs can directly trigger histamine release from mast cells and its potential to exacerbate allergic symptoms. This effect is due to histamine's ability to affect the physiology and pathology of a wide range of cells via corresponding receptors that are widely expressed on airway and vascular muscle cells, hepatocytes, chondrocytes, endothelial cells, epithelial cells, neutrophil, eosinophils, monocytes, macrophage, dendritic cells, and T and B cells [4]. We found that TiO_2 _NPs can significantly induce histamine release from RBL-2H3 cells in a dose dependent manner (Figure 5A). Our data is corroborated by evidence documenting more severe TiO_2 _NP-induced inflammation with elevated release of inflammatory cytokines than that of TiO_2 _of micrometric dimensions [31,32]. The TiO_2 _NP-elicited mast cell degranulation also appeared to be a Ca^2+^-dependent process, as indicated by a major attenuation in histamine secretion when pretreated with BAPTA (Figure 5B). The importance of Ca^2+ ^in stimulus-secretion coupling mechanisms is well documented [33,34]. We and others have reported that NPs increase [Ca^2+^]_C _prior to cellular exocytosis [17,35]. As TiO_2 _concentrations increased from 0.1 to 1 mg/ml, a sustained elevation in [Ca^2+^]_C _was observed (Figure 2A). Our data demonstrated that [Ca^2+^]_C _increased as a function of TiO_2 _concentration and coincided with the measurements of histamine secretion (Figure 5A). The TiO_2 _NPs used in our study consisted of a mixture of anatase/rutile crystalline forms which have been shown to impose greater cellular toxicity [22]. Although phase composition, surface chemistry and purity may play important roles in influencing nanotoxicity, dependence on additional physical parameters such as particle size, surface area and concentration have all been reported [20,21,36-38]. However, the mechanism by which TiO_2 _NPs trigger intracellular mast cell Ca^2+ ^signaling is not clear.

Previous experimental evidence suggests that ultrafine carbon black and ZnO NPs can induce extracellular Ca^2+ ^influx through activated voltage-gated Ca^2+ ^channels [35,39]. The question of whether TiO_2 _NPs can trigger a similar process is unclear. Our experiments confirmed that [Ca^2+^]_C _failed to increase significantly when cells were treated with TiO_2 _NPs in Ca^2+^-free Hanks' solution (Figure 2B) or blocked with CdCl_2 _(a general Ca^2+ ^channel blocker) (Figure 2C) [17]. We then demonstrated that TiO_2 _NPs can activate L-type voltage-gated Ca^2+ ^channels on mast cells, allowing extracellular Ca^2+ ^influx into the cytosol (Figure 2D and 2E). The ROS generated in response to NP exposure has been attributed to the opening of membrane Ca^2+ ^channels [17,35,39,40]. Our data revealed that TiO_2 _NPs elevated cytosolic ROS levels (Figure 3A) and that the involvement of oxidative stress could modulate intracellular Ca^2+ ^homeostasis by activating L-type Ca^2+ ^channels (Figure 3B). Supporting our results, Hussain and colleagues also reported that TiO_2 _NPs can exert oxidative effects under abiotic conditions [23]. Other studies have shown that TiO_2 _NPs increase intracellular ROS production in various cells by causing mitochondrial injuries, such as impaired mitochondrial membrane permeability, a decrease in mitochondrial potential, mitochondrial respiratory dysfunction and downregulation of SOD (superoxide dismutase) and GSH (glutathione) levels [41,42]. ROS are thought to affect Ca^2+ ^signaling possibly by oxidation of thiol groups of membrane channels and changing the intra-molecular bonding of proteins and lipids [43,44]. Moreover, it has been suggested that ROS can impact membrane Ca^2+ ^channels and Ca^2+ ^binding proteins [43]. In addition to ROS formation, direct damage to cell membrane integrity by lipid peroxidation may be an alternative route for extracellular Ca^2+ ^entry [22,39]. Co-administration of TiO_2 _NPs and fluorescent calcein dye resulted in an augmented calcein permeation ratio (Figure 2F). The calcein data corroborates with previous publications showing that TiO_2 _NPs can perturb the lipid bilayer, perhaps by forming transient pores that may account for the portion of Ca^2+ ^increase that could not be completely abolished by blocking L-type Ca^2+ ^channels [17,45].

An initial upsurge in [Ca^2+^]_C _from extracellular influx is usually relayed by a secondary Ca^2+ ^release from internal organelles [46]. However, the question of how TiO_2 _NPs transduce Ca^2+ ^signals in mast cells involving common secondary messengers remains unanswered. Heretofore, we demonstrated that TiO_2 _NPs could stimulate PLC and IP_3 _receptor activities in mast cells, resulting in secondary amplification of cytosolic Ca^2+^. Data from Figure 4A-C showed that cells incubated with U73122, 2-APB and Xestospongin notably inhibited TiO_2 _NP-triggered Ca^2+ ^rise and histamine secretion (Figure 5B). Activation of PLC can be explained by the stimulation from ROS [47,48]. Alternatively, a localized increase in [Ca^2+^]_C _has been shown to trigger PLC, thereby generating IP_3 _and DAG [49]. Collectively, these results point toward the contribution from ER Ca^2+ ^stores. Figure 4D revealed that depleting the ER internal store with Thapsigargin significantly diminished TiO_2 _NPs stimulated cytosolic Ca^2+ ^increase and subsequent histamine secretion (Figure 5B). Our data indicates that the ER dependent IP_3_-IP_3 _receptor pathway is critically involved in amplifying (or sustaining) the initial cytosolic Ca^2+ ^rise and subsequent histamine release.

## Conclusion

Our findings revealed a new mechanism depicting how TiO_2 _NPs can potentially exacerbate many allergic inflammatory responses and perhaps non-allergic inflammatory disorders. Mast cell exposure to TiO_2 _NPs can activate membrane L-type Ca^2+ ^channels, induce ROS production and stimulate PLC activity. Influx of extracellular Ca^2+ ^raises [Ca^2+^]_C_, and when coupled with the IP_3_-IP_3 _receptor pathway, can trigger the release of ER resident Ca^2+ ^and subsequent histamine secretion. These results suggest that mast cell degranulation of histamine may be significantly augmented and intensified in NP exposed tissues with or without IgE antibody-based sensitization. This model may also provide a new explanation for chronic inflammatory diseases elicited by airborne PM. The inhaled NPs can circulate and accumulate in various organs thereby elevating the risks of activating mast cell degranulation in other tissues [25,50,51]. As a result, NP exposure may worsen the mast cell associated inflammatory symptoms involving arthritis, atherosclerosis and coronary diseases [52-54]. Finally, our results suggest that a new immunoregulatory system may be considered since NPs can directly trigger inflammatory mediators, thereby bypassing traditional immuno-stimulation by allergens.

## Methods

### Culture of RBL-2H3 Mast Cells

The RBL-2H3 rat mast cell line (ATCC, Manassas, VA, USA) is a widely-used mast cell model that responds to stimuli by secreting histamine and other mediators [55]. RBL-2H3 cells elicit a potent immune allergic response following crosslinking of their IgE-bound FcεRI by multivalent allergens [55]. Cells were cultured in 15 cm cell culture plates (VWR, CA, USA) in MEM medium (Invitrogen, CA, USA) supplemented with L-glutamine, 1% penicillin/streptomycin and 10% heat inactivated fetal bovine serum (FBS) (Invitrogen, CA, USA). Cultures were incubated in a humidified incubator at 37°C/5% CO_2_. Cell counts were performed using trypan blue (Sigma-Aldrich, MO, USA) exclusion and a Bright-Line haemocytometer.

### Nanoparticles and Characterization

A mixture of anatase and rutile forms of ultrafine titanium (IV) dioxide (< 100 nm diameter by Brunauer Emmett Teller (BET) method, purity of 99.5% trace metals basis) (Cat No. 634662) (Sigma-Aldrich, MO, USA) was used in this study because this form has been shown to result in more severe cellular injuries [22]. The TiO_2 _NPs have a primary crystalline size of 100 nm (maximum), specific surface area of 46.3 m^2^/g (as determined by BET), equivalent spherical diameter of 100 nm (maximum) and a trace metallic impurities of 1000 ppm (maximum) (information from Sigma). All TiO_2 _NP samples were reconstituted in Hanks' buffer (Invitrogen, CA, USA) and sonicated for approximately 1 minute immediately before usage. The concentrations used were 1 mg/ml, 0.75 mg/ml, 0.5 mg/ml, 0.25 mg/ml, and 0.1 mg/ml. All sizes of NP suspension were independently confirmed using homodyne dynamics laser scattering (DLS) as described in the previous study [17]. Morphology and size of TiO_2 _NP powder were determined by transmission electron microscope (JEOL JEM-2010 HRTEM, MA, USA) and analyzed using an analysisPRO (Olympus, PA, USA).

### Cell Preparation

Cells were seeded at 1 × 10^5 ^cells per well in a 24-well plate, and incubated for 24 hrs in MEM medium supplemented with 10% FBS. Following 24 hr incubation, the MEM medium was removed from the cells and the culture was rinsed with Hanks' solution twice before use.

### Measurement of cytosolic Ca^2+ ^concentrations induced by TiO_2 _exposure

All experiments were performed in dark conditions. The cells were loaded with a Rhod-2 AM dye (1 μM) (K_d _= 570 nM, λ_Ex _= 552 nm and λ_Em _= 581) (Invitrogen, CA, USA) for 45 minutes. After the dye loading, the cells were rinsed and incubated with either normal Hanks' or Ca^2+^-free Hanks' solution, and treated with the appropriate TiO_2 _NP concentrations. All Ca^2+ ^signaling experiments were carried out on a thermoregulated stage at 37°C mounted on a Nikon microscope (Nikon Eclipse TE2000- U, Tokyo, Japan). RBL-2H3 cells were then incubated with cadmium chloride (200 μM; Sigma-Aldrich, MO, USA) to block the membrane Ca^2+ ^channels [17], followed by TiO_2 _NP stimulation. To test the interaction between TiO_2 _and L-type membrane Ca^2+ ^channels, nifedipine (10 μM; Sigma-Aldrich, MO, USA) and verapamil (100 μM; Sigma-Aldrich, MO, USA), L-type Ca^2+ ^channel blockers [17,39] were applied to RBL-2H3 cells, independently, prior to the exposure of TiO_2_. Antioxidant N-acetylcysteine (NAC, 250 μM; Sigma-Aldrich, MO, USA) was also added to RBL-2H3 cells to study the involvement of reactive oxygen species (ROS) [17,39], possibly generated as a result of TiO_2 _stimulation, and the activation of Ca^2+ ^channels. Thapsigargin (100 nM; Sigma-Aldrich, MO, USA) was used to deplete the ER Ca^2+ ^content in order to investigate the contribution of the internal ER Ca^2+ ^pool [17,56]. In order to test the involvement of secondary messenger molecules in intensifying a Ca^2+ ^response, U73122 (PLC blocker) (10 μM; Sigma-Aldrich, MO, USA), Aminoethoxydiphenyl borate (2-APB) (50 μM; Sigma-Aldrich, MO, USA) and Xestospongin C (IP_3 _receptor blockers) (20 μM; VWR, CA, USA) were pre-incubated with cells prior to TiO_2 _NP exposure [56,57].

### Calcein dye leakage measurements

RBL-2H3 cells were seeded at a density of 1 × 10^5 ^cells per well in a 24-well plate and cultured for 24 hrs. TiO_2 _prepared with calcein fluorescent dye (50 μM) (Invitrogen, CA, USA) in Hanks' buffer was incubated with the cells for 5 minutes at 37°C. Calcein is a biological inert green-fluorescent molecule of a molecular mass of 623 Daltons and an estimated molecular radius of 0.6 nm [17]. The TiO_2 _NP solution containing calcein dye was then removed and the cells were rinsed twice with PBS to remove any possible remnants of the dye. Subsequently, the cells were loaded with Hoechst (10 μM) (Sigma-Aldrich, MO, USA), a fluorescent nucleus dye, for 5 minutes at 37°C, and were then rinsed thoroughly [17]. Fresh Hanks' solution was added into each well before taking fluorescent images of calcein and Hoechst loaded cells with a Nikon fluorescence microscope. Afterwards, a percentage of calcein loaded cells against total number of cells, as determined by the Hoechst dye, was calculated for each of the TiO_2 _NP concentrations used in the experiment.

### Intracellular reactive oxygen species (ROS) production

ROS production was evaluated by fluorescence microscopy using oxidation of CM-H_2_DCFDA dye (Invitrogen, CA, USA). The cells (1 × 10^5 ^cells/well) were cultured for 24 hrs before being rinsed with PBS solution. Hanks' buffer, containing TiO_2 _NPs at concentrations ranging from 0-0.75 mg/ml, was then incubated with the cells for 15 minutes in 37°C followed by PBS washing and loading with 2 μM CM-H_2_DCFDA dye for 30 minutes. Fluorescent images of ROS generated in cells were captured and analyzed by calculating the ratio of increase in fluorescent intensity between TiO_2 _NP treatment and control groups.

### Histamine detection with enzyme linked immunosorbent assay (ELISA) preparation

The cells were seeded at a density of 1 × 10^5 ^cells density in a 24-well plate and cultured for 24 hrs. RBL-2H3 cells were then rinsed with PBS and pre-treated with BAPTA-AM (50 μM) (Invitrogen, CA, USA), Thapsigargin (SERCA pump inhibitor), U73122, 2-APB, or Xestospongin for 20 minutes in the same manner. Cells were stimulated for 5 minutes with the appropriate TiO_2 _NP concentrations (0-0.75 mg/ml) which were prepared in PBS. The histamine-containing supernatant was collected and centrifuged to remove any remaining TiO_2 _NPs. The supernatant was then incubated in a 96-well plate overnight at 4°C. The rest of the assay was carried out in accord to Neogen ELISA instructions pertaining to the histamine kit (Neogen Corp, MI, USA).

### Image Analysis

After staining the treated cells, image analysis was performed with an inverted Nikon Eclipse TE2000-U fluorescent microscope. Each photo was taken at a magnification of 200 × and analyzed using SimplePCI (Compix Inc., Imaging Systems, Sewickle, PA, USA). The data shown is a representative of Ca^2+ ^signals of more than 200 cells and the experiments were conducted independently for at least 3 times.

### Statistical Analysis

The data was presented as means ± SD. Each experiment was performed independently at least three times. Statistical significance was determined using a Student's t-test analysis with p values < 0.05 (GraphPad Prism 4.0, GraphPad Software, Inc., San Diego, CA, USA).

## Competing interests

The authors declare that they have no competing interests.

## Authors' contributions

EYC, MG, YCW: Designed research, conducted experiments, analyzed and interpreted data. EYC, MG, YCW, AJM, CSC and WCC: wrote, reviewed and revised the manuscript. All authors have read and approved the final manuscript.
